# Contamination of *Vibrio parahaemolyticus* in crayfish for sale

**DOI:** 10.3389/fmicb.2024.1388658

**Published:** 2024-08-14

**Authors:** Kui Wu, Dazhao Zou, Yongyan Long, Lin Xue, Shufen Shuai, Feiyan Tian, Mei Li, Guoyin Fan, Yangyun Zheng, Xiangrong Sun, Wei Wang, Li Wang, Xiansheng Ni, Xiaoling Zhang, Yibing Fan, Hui Li

**Affiliations:** ^1^The Collaboration Unit for State Key Laboratory of Infectious Disease Prevention and Control, Jiangxi Provincial Health Commission Key Laboratory of Pathogenic Diagnosis and Genomics of Emerging Infectious Diseases, Nanchang Center for Disease Control and Prevention, Nanchang, China; ^2^Jiangxi Provincial Key Laboratory of Preventive Medicine, School of Public Health, Jiangxi Medical College, Nanchang University, Nanchang, China; ^3^Jiangxi Provincial Center for Agricultural Technical Extension, Nanchang, China; ^4^Donghu District Center for Disease Control and Prevention, Nanchang, China

**Keywords:** *Vibrio parahaemolyticus*, crayfish, contamination, adsorption, aquatic products markets

## Abstract

Crayfish (*Procambarus clarkii*) are economically important freshwater crustaceans. With the growth of the crayfish industry, the associated food-safety risks should be seriously considered. Although *Vibrio parahaemolyticus* is commonly recognized as a halophilic foodborne pathogen associated with seafood, it has been found to be a major pathogen in crayfish-associated food poisoning cases. In this study, the *V. parahaemolyticus* contamination level in crayfish production-sale chain was investigated using crayfish and environmental samples collected from crayfish farms and markets. Serious *V. parahaemolyticus* contamination (detection rate of 66%) was found in the entire crayfish production-sale chain, while the *V. parahaemolyticus* contamination level of the market samples was extremely high (detection rate of 92%). The *V. parahaemolyticus* detection rate of crayfish surface was similar to that of whole crayfish, indicating that crayfish surface was important for *V. parahaemolyticus* contamination. The simulation experiments of crayfish for sale being contaminated by different *V. parahaemolyticus* sources were performed. All the contamination sources, containing *V. parahaemolyticus-*positive tank, water, and crayfish, were found to be efficient to contaminate crayfish. The crayfish tank displayed the most significant contaminating role, while the water seemed to inhibit the *V. parahaemolyticus* contamination. The contamination extent of the crayfish increased with the number of *V. parahaemolyticus* cells the tank carried and the contact time of the crayfish and the tank, but decreased with the time that the crayfish were maintained in the water. It was also confirmed that the crayfish surface was more susceptible to *V. parahaemolyticus* contamination than the crayfish intestine. Furthermore, the adsorption of *V. parahaemolyticus* onto the crayfish shell was analyzed. Over 90% of the *V. parahaemolyticus* cells were adsorbed onto the crayfish shell in 6 h, indicating a significant adsorption effect between *V. parahaemolyticus* and the crayfish shell. In conclusion, within a water-free sale style, the fresh crayfish for sale in aquatic products markets uses its shell to capture *V. parahaemolyticus* cells from the *V. parahaemolyticus-*abundant environments. The *V. parahaemolyticus* contamination in crayfish for sale exacerbates the crayfish-associated food-safety risk. This study sheds light on *V. parahaemolyticus* control and prevention in crayfish industry.

## 1 Introduction

*Vibrio parahaemolyticus* is a seafood-associated foodborne pathogen (Dutta et al., 2021). The pathogen is a moderately halophilic bacterium typically living in saline environments such as salt lakes and seas. People who consume seafood contaminated by *V. parahaemolyticus* run the risk of a series of food poisoning syndromes (Raszl et al., 2016; Xu et al., 2022). In the United States, *V. parahaemolyticus* is one of the main pathogens involved in reported vibriosis, where the incidence in 2010 was 0.28 to 0.42 per 100,000 population (Newton et al., 2012). In China, *V. parahaemolyticus* is also an important pathogen causing infectious diarrhea (Han et al., 2022). It is estimated that there are approximately 4.951 million cases of food poisoning cases induced by *V. parahaemolyticus* each year in China (Mao et al., 2013).

The total detection rate of *V. parahaemolyticus* contamination in China aquatic products was 32.20% (Pang et al., 2020). However, as a halophilic microorganism (Namadi and Deng, 2023), *V. parahaemolyticus* is often detected in seafood. Even though there are cases of *V. parahaemolyticus* being found in freshwater products (Pei et al., 2016), this is generally not regarded as a high risk. However, in recent years, *V. parahaemolyticus* has been increasingly detected in crayfish (*Procambarus clarkii*), one of the most economically important freshwater products in China (Dong et al., 2016; Long et al., 2023). Unsurprisingly, crayfish consumption can lead to serious *V. parahaemolyticus* infection (Bean et al., 1998; Sun et al., 2012). China has the world largest crayfish industry. According to a recent study (Yu et al., 2023), the output value of Chinese crayfish industry in 2022 was estimated to be 458 billion yuan, with a crayfish farming area of 28 million mu (one mu equals 667 m^2^) and a crayfish catch of 2.89 million tons. With the large amount of crayfish production and consumption, food-safety risk in the crayfish industry needs to be seriously considered, especially *V. parahaemolyticus-*associated contamination.

In China, consumers prefer fresh crayfish rather than processed crayfish products. Thus, the microbial hygiene indicators of fresh crayfish in markets are relatively important. In this study, we collected crayfish and crayfish-associated environmental samples from crayfish farms and aquatic products markets to investigate the *V. parahaemolyticus* contamination level of the crayfish industry. We found that the *V. parahaemolyticus* contamination of the crayfish sale stage was quite serious (with the *V. parahaemolyticus* detection rate of 92%). Both the market environmental samples and the for-sale crayfish samples showed extremely high *V. parahaemolyticus* contamination levels (with the *V. parahaemolyticus* detection rate of 95% for tank swabs and 91% for for-sale crayfish samples). We also simulated the process of different *V. parahaemolyticus* sources contaminating the crayfish for sale in aquatic products markets, and found that the *V. parahaemolyticus-*positive (VP^+^) crayfish container was the most important contamination source. In addition, we have confirmed that the *V. parahaemolyticus* can adsorb onto crayfish shells, which enhances the *V. parahaemolyticus* contamination in crayfish for sale.

## 2 Materials and methods

### 2.1 Sample collection

In this study, three types of samples comprising crayfish, water, and tank swabs were collected from crayfish farms, wholesale and retail aquatic products markets during the period from May to September in 2024. The crayfish farms for sample collection were located in 10 counties (Jinxian, Xinjian, Yongxiu, Duchang, Pengze, Yugan, Wannian, Yujiang, Jishui, and Xingan counties) in a crayfish production area around Poyang Lake in Jiangxi Province, China. The wholesale and retail markets for sample collection were located in Nanchang, the provincial capital city that features a large consumption of crayfish. The sampling information was listed in Supplementary Table S1.

The crayfish samples were bought from market stalls or crayfish farms. Each crayfish sample weighed approximately 500 g and was composed of 20–30 fresh crayfish individuals. The crayfish samples were packaged separately in puncture-resistant sterile plastic bags and immediately sent to our laboratory for detection of *V. parahaemolyticus*. Water samples were collected from the crayfish farms. The water sample collection method referred to the Cholera Control Manual (6th edition) (Xiao, 2013), using a sterile water sampler to collect 450 mL of water from 20 cm under the surface in the crayfish farming pools and adding the water to a sterilized 500-mL glass bottle containing 50 mL 10× 3% NaCl alkaline peptone water (3% NaCl APW, Huankai, China; Peptone 10 g/L, NaCl 30 g/L, pH 8.5 ± 0.2). The glass bottles containing the water samples were transported to our laboratory and incubated at 37°C for 18–24 h. The tank swab samples were collected from the crayfish stalls in the markets. When collecting the tank swabs, the inner side surface of the crayfish tank was swabbed (10 cm × 10 cm) using a sterile swab, and then the swab was placed into a sterilized tube containing 9 mL of 3% NaCl APW. The tubes with swab samples were transported to the laboratory and incubated at 37°C for 18–24 h. In total, 154 samples comprising 75 crayfish samples from markets, 30 crayfish samples from crayfish farms, 29 crayfish farming water samples from crayfish farms, and 20 crayfish tank swabs from markets were collected to detect *V. parahaemolyticus* contamination.

### 2.2 *Vibrio parahaemolyticus* detection

The samples collected from wholesale and retail markets in Nanchang were immediately transported to our lab at room temperature in less than 2 h, the samples collected from crayfish farms which located into different cities in Jiangxi Province were transported to our lab at 4°C in less than 24 h. Upon arrival at the lab, the crayfish samples were treated according to the standard method GB/T 4789.7–2013 (National Health and Family Planning Commission of the People's Republic of China, 2013). In brief, 3–5 crayfish were randomly chosen, washed twice with sterilized water, and homogenized or cut into pieces under aseptic conditions. Twenty-five grams of the crayfish homogenate was added to a flask with 225 mL 3% NaCl APW to enrich the target microorganisms by incubation at 37°C for 18–24 h.

*Vibrio parahaemolyticus* isolation and identification from the enrichment cultures of crayfish samples, water samples, and swab samples was based on Xu’s method (Xu et al., 2014). The enrichment cultures were streak-inoculated on thiosulfate citrate bile salts sucrose agar (TCBS, Huankai, China). The TCBS plates were cultured at 37°C overnight. The typical *V. parahaemolyticus* strain colony on TCBS was circular, translucent, smooth, and green. If possible, to avoid false-negative results, at least three suspected *V. parahaemolyticus* strains were chosen to inoculate to CHROMagar™ Vibrio chromogenic medium (CHROMagar, France). The chromogenic medium plate was incubated at 37°C for 18–24 h, and the mauve strains growing on the chromogenic medium were recognized as *V. parahaemolyticus* and stored at −20°C.

Some crayfish samples from markets were also used for *V. parahaemolyticus* detection of the crayfish surface (CS) and the crayfish intestine (CI). The procedure was as follows. Two crayfish were randomly chosen from a crayfish sample. A sterile swab was used to wipe the surfaces of the two crayfish, then the CS swab was treated with the same detection process as the container swabs. After wiping with the swab, the two crayfish were placed in a beaker containing 200 mL of 75% ethanol for 2 min to disinfect the body surface. The intestines were removed from the surface-disinfected crayfish and placed into sterilized tubes with 9 mL of 3% NaCl APW. The subsequent detection process for the CI sample was the same as for the swabs.

### 2.3 Simulation of *Vibrio parahaemolyticus* contamination in crayfish

Laboratory simulation tests of *V. parahaemolyticus* contamination were performed in order to determine how the crayfish in the markets were contaminated with *V. parahaemolyticus*. The *V. parahaemolyticus* 2023-M55-1 isolated from crayfish was used as the contamination strain for the simulations. Before the simulations, the *V. parahaemolyticus* strain 2023-M55-1 was rejuvenated overnight with 3% NaCl APW at 37°C. The *V. parahaemolyticus* culture was tenfold serially diluted using sterilized 3% NaCl saline, and the *V. parahaemolyticus* concentration in the culture was determined by a plate counting method with 3% NaCl nutrient agar (Huankai, China). The *V. parahaemolyticus-*negative (VP^−^) crayfish (10–15 g per crayfish) used as the contamination targets were supplied by a crayfish farm in Yongxiu county and were verified to be VP^−^ by the *V. parahaemolyticus* detection methods described in section 2.2. A sterilized four-liter plastic tank (18 cm in length, 14 cm in width, and 15 cm in height) was used as the container where the *V. parahaemolyticus* contamination occurred in each laboratory simulation. Three contamination factors of a *V. parahaemolyticus*-positive (VP^+^) tank, VP^+^ water, and VP^+^ crayfish, situations that the crayfish would encounter in the sale process, were considered in the simulation tests. Each of the contamination simulation tests was performed at 25°C.

To prepare a VP^+^ tank, 500 μL of *V. parahaemolyticus* suspension with an appropriate bacterial concentration was added to a tank. After that, the liquid was uniformly spread on the bottom and inner walls of the tank by shaking the tank several times. Then, the tank was kept at 37°C for 0.5 h to dry the *V. parahaemolyticus* liquid, and thus a VP^+^ tank had been prepared. Twenty-four robust VP^−^ crayfish were placed into the VP^+^ tank to initiate the simulated contamination. At different time points (0.5, 2, 4, 8, and 24 h), four crayfish were randomly chosen for *V. parahaemolyticus* detection.

For simulated contamination by VP^+^ water, 500 μL of an appropriate *V. parahaemolyticus* suspension was added to 500 mL of sterile water in a tank, and 24 robust VP^−^ crayfish were immediately soaked in the VP^+^ water. When simulating the scenario in which crayfish were preserved in water for sale, the crayfish were continuously soaked in the VP^+^ water until they were chosen for *V. parahaemolyticus* detection. When simulating the scenario in which crayfish were washed with water before sale, the crayfish were placed in the VP^+^ water for only 0.5 h, and then the VP^+^ water was removed from the tank. At 0.5, 2, 4, 8, and 24 h after the crayfish contacting with the VP^+^ water, four crayfishes were randomly chosen for *V. parahaemolyticus* detection. Considering that the *V. parahaemolyticus* strain could die in fresh water, 1 mL of the VP^+^ water was removed to a 9 mL 3% NaCl APW tube for *V. parahaemolyticus* detection at the appropriate time. The VP^+^ water in a tank without crayfish was used as the control.

For simulated contamination by VP^+^ crayfish, the VP^−^ crayfish were soaked in the VP^+^ water with a *V. parahaemolyticus* concentration of 5 × 10^4^ colony forming units per milliliter (CFU/mL) for 8 h at 25°C to prepare the VP^+^ crayfish. Four VP^+^ crayfish were washed with sterilized water, marked with a red string tied to their chelipeds, and moved to a plastic tank containing 20 VP^−^ crayfish. After an appropriate time (0.5, 2, 4, 8, and 24 h), four of the original VP^−^ crayfish were randomly chosen for *V. parahaemolyticus* detection. The VP^+^ crayfish shell was also prepared and used to contaminate VP^−^ crayfish with the same methods. The crayfish shells were bought from an agricultural product processing factory in Jiujiang City.

### 2.4 *Vibrio parahaemolyticus* detection for the contamination simulations

The *V. parahaemolyticus* contamination in the simulation experiments was verified by an enrichment-PCR method. First, the crayfish, the CS, and the CI samples were added to 3% NaCl APW for *V. parahaemolyticus* enrichment using the method described in section 1.2. DNA extraction was performed with the overnight cultures using a DNA purification kit (Tiangen, China). The DNA samples were used for a PCR assay based on the standard method SN/T1869-2007 (General Administration of Quality Supervision, Inspection and Quarantine of the People's Republic of China, 2007). The samples generating a PCR product of 450 bp were recognized as PCR-positive. Each simulation experiment was repeated three times. The contamination result for each simulation condition was determined to be positive only there were at least two PCR-positives in the three repeats.

### 2.5 The adsorption tests of *Vibrio parahaemolyticus* onto the crayfish shell

Kaneko’s method (Kaneko and Colwell, 1975) was used to analyze the adsorption of *V. parahaemolyticus* onto the crayfish shell. Before use, the crayfish shell was washed with 0.5% NaCl saline and filtered through three-layer sterile gauze to remove the soluble components and the filterable particles. Then, 5 g of crayfish shell was added to a 300-mL flask and suspended with 100 mL of 0.5% NaCl saline. The overnight culture of *V. parahaemolyticus* 2023-M55-1 was centrifuged and then resuspended in 100 mL of 0.5% NaCl saline. An appropriate volume of the *V. parahaemolyticus* suspension was added to the flask with crayfish shell suspension. The final *V. parahaemolyticus* concentration of the mixture was about 10^6^ CFU/mL. The mixture was gently agitated (100 r/min) at 20°C. At the appropriate time, a portion of the mixture (about 2 mL) was taken up and filtered through three-layer sterile gauze to remove the crayfish shell. The filtrate was used to determine the number of the *V. parahaemolyticus* cells with a plate-counting method. As the crayfish shell was not bacteria-free initially, the TCBS media was employed in the plate-counting test to avoid interference from other microorganisms. An 0.5% NaCl saline solution without any crayfish shell was used as the control in the adsorption experiment.

## 3 Results

### 3.1 The *Vibrio parahaemolyticus* contamination levels in the crayfish production-sale chain

The crayfish and environmental samples from crayfish farms, wholesale and retail markets were tested in this study in order to determine the greatest risk point for *V. parahaemolyticus* contamination in the crayfish production-sale chain (Table 1). One hundred and one out of the tested 154 samples were VP^+^, indicating serious *V. parahaemolyticus* contamination in the crayfish production-sale chain. Of the 37 crayfish samples collected from wholesale markets, 34 were VP^+^. Similarly, *V. parahaemolyticus* was also isolated from 34 out of the 38 retail market samples. The total VP^+^ rate of the for-sale crayfish samples was quite high at 90.7%, irrespective of the crayfish source. In contrast, the crayfish samples collected from farms displayed a much lower VP^+^ rate, only 13 of the 30 samples were VP^+^, indicating that more serious *V. parahaemolyticus* contamination occurred in the markets. Furthermore, investigation of the crayfish-associated environmental samples showed that 19 out of 20 tank swabs from the crayfish stalls were VP^+^, while one out of 29 crayfish farming water samples was VP^+^. The extremely high VP^+^ rate (95.0%) of the crayfish tanks in the markets was an important reason for the high contamination level in the crayfish for sale. The crayfish farm water was generally *V. parahaemolyticus-*free (1 VP^+^ in total 29 water samples) and thus may not play a significant role in *V. parahaemolyticus* contamination. Selling in markets appears to be the highest risk point for *V. parahaemolyticus* contamination in the crayfish production-sale chain.

**Table 1:** *Vibrio parahaemolyticus* detection results for the samples from the crayfish production-sale chain.

Sample source	Wholesale markets		Retail markets	Crayfish farms	
Sample type	Tank swap	Crayfish	Crayfish	Crayfish	Water
Sample numbers	20	37	38	30	29
VP^+(a)^ samples	19	34	34	13	1
VP^+^ rates	95.0%	91.9%	89.5%	43.3%	3.3%
		90.7%^(b)^			

^(a)^VP^+^ means *V. parahaemolyticus* positive.^(b)^Total VP^+^ rate of the crayfish samples collected from markets.

### 3.2 The *Vibrio parahaemolyticus* contamination levels in different parts of the crayfish for sale

To examine the *V. parahaemolyticus* contamination in greater detail, 30 of the 75 market crayfish samples were used to detect *V. parahaemolyticus* with different parts (the CS, the CI, and the whole crayfish). The results are shown in Table 2. Using the whole crayfish as the target, all 30 samples were VP^+^. With the CS and the CI as the targets, 25 and seven of the 30 crayfish samples were VP^+^, respectively. In the seven CI-VP^+^ samples, only one was VP^−^ for the CS; the other six samples were VP^+^ for both CS and CI samples. Thus, the *V. parahaemolyticus* contamination level of the crayfish surface was close to that of the whole crayfish, while the intestine of the crayfish was relatively uncontaminated. As the crayfish surface could be more easily exposed to the *V. parahaemolyticus* source, it may be an important target for *V. parahaemolyticus* contamination.

**Table 2:** *Vibrio parahaemolyticus* detection results for different parts of the crayfish collected from markets.

Detection target	Whole crayfish	Crayfish surface	Crayfish intestine
Detection numbers	30	30	30
VP^+(a)^ numbers	30	25	7

^(a)^VP^+^ means *V. parahaemolyticus* positive.

### 3.3 The *Vibrio parahaemolyticus* contamination by the VP^+^ tank

Fresh crayfish in the markets are usually placed in various plastic containers without water. In the simulation tests, VP^+^ tanks with different original numbers of *V. parahaemolyticus* were prepared and used to hold the VP^−^ crayfish. We found that the crayfish could be easily contaminated by the VP^+^ tanks (Table 3). Even though the number of *V. parahaemolyticus* on the inner surface of the tank was as low as 2 CFU/cm^2^, the VP^−^ crayfish would become VP^+^ within 2 h. When the number of *V. parahaemolyticus* was increased to 2 × 10^1^ CFU/cm^2^, the crayfish became VP^+^ within 0.5 h. The detection results for the CS samples were similar to those for the whole crayfish. However, compared to the whole crayfish, successful *V. parahaemolyticus* contamination on the crayfish surface needed more initial bacteria and a longer contact time in the VP^+^ tanks. When the number of *V. parahaemolyticus* reached 2 × 10^2^ CFU/cm^2^, the CI samples were VP^+^ at 8 h and 24 h. When the number of *V. parahaemolyticus* was 2 × 10^3^ CFU/cm^2^, the time for the CI samples to become VP^+^ was shortened to 4 h. Thus, for the crayfish tank, the more *V. parahaemolyticus* and the longer the time the crayfish were exposed, the more serious the level of *V. parahaemolyticus* contamination.

**Table 3:** Results of the simulation tests of crayfish contaminated by the tank carrying *V. parahaemolyticus*.

Original numbers of *V. parahaemolyticus* in the tank (CFU/cm^2^)	Contact time between the crayfish and the tanks (h)														
	Whole crayfish					Crayfish surface					Crayfish intestine				
	0.5	2	4	8	24	0.5	2	4	8	24	0.5	2	4	8	24
2 × 10^0^	−	+	+	+	+	−	−	−	+	−	−	−	−	−	−
2 × 10^1^	+	+	+	+	+	−	+	+	+	+	−	−	−	−	−
2 × 10^2^	+	+	+	+	+	+	+	+	+	+	−	−	−	+	+
2 × 10^3^	+	+	+	+	+	+	+	+	+	+	−	−	+	+	+

–: *V. parahaemolyticus* negative. +: *V. parahaemolyticus* positive.

### 3.4 The *Vibrio parahaemolyticus* contamination by the VP^+^ water

Although the crayfish were generally sold without water, the crayfish for sale were sometimes stored in water in some markets. The crayfish might be contaminated by the VP^+^ water. In the contamination simulations, we found that it was difficult to contaminate the crayfish by the VP^+^ water, (Table 4). When the *V. parahaemolyticus* concentration in the VP^+^ water was 5 × 10^2^ CFU/mL, the whole crayfish samples became VP^+^ at 2, 4, and 8 h, but neither the CS nor the CI samples were VP^+^ at any of the testing times. When the *V. parahaemolyticus* concentration was increased to 5 × 10^3^ CFU/mL, the whole crayfish samples were VP^+^ at 0.5, 2, 4, and 8 h, and the CS samples were VP^+^ only at 2 h. When the *V. parahaemolyticus* concentration reached 5 × 10^4^ CFU/mL, the whole crayfish samples were VP^+^ at all testing times, and the CS samples were VP^+^ at 0.5, 2, 4, and 8 h. Irrespective of the *V. parahaemolyticus* concentration in the VP^+^ water, the CI samples were VP^−^ at all testing times, meaning that the crayfish intestine was difficult to become contaminated. Although the VP^+^ rate of the crayfish samples increased with the *V. parahaemolyticus* concentration, the time of the crayfish in the VP^+^ water had a negative effect on the *V. parahaemolyticus* contamination. The VP^+^ rate of the crayfish samples held in VP^+^ water for 24 h was lower than those held for 4 and 8 h. In addition, the VP^+^ rates of the crayfish samples held in VP^+^ water for 4 and 8 h were also lower than those held for 2 h.

**Table 4:** Results of the simulation tests of crayfish contaminated by water carrying *V. parahaemolyticus*.

Original numbers of *V. parahaemolyticus* in the water (CFU/mL)	Holding time for crayfish in the water (h)														
	Whole crayfish					Crayfish surface					Crayfish intestine				
	0.5	2	4	8	24	0.5	2	4	8	24	0.5	2	4	8	24
5 × 10^2^	−	+	+	+	−	−	−	−	−	−	−	−	−	−	−
5 × 10^3^	+	+	+	+	−	−	+	−	−	−	−	−	−	−	−
5 × 10^4^	+	+	+	+	+	+	+	+	+	−	−	−	−	−	−

–: *V. parahaemolyticus* negative. +: *V. parahaemolyticus* positive.

It is generally agreed that *V. parahaemolyticus* is a halophilic bacterium and that NaCl is necessary for its survival and reproduction. Along with the simulation experiments, the survival of the *V. parahaemolyticus* strain in water was also tested. The results showed that the pure water without salt ions decreased the survival of *V. parahaemolyticus* (Table 5). When the *V. parahaemolyticus* concentration of the VP^+^ water was equal to or less than 5 × 10^3^ CFU/mL, the water samples could not be detected as being VP^+^ after 0.5 h. Even if the original *V. parahaemolyticus* concentration was increased to 5 × 10^4^ CFU/mL, the water samples were VP^+^ only at 0.5 and 2 h. The longer *V. parahaemolyticus* was maintained in fresh water, the lower the probability of testing VP^+^. Interestingly, the crayfish in the water enhanced the survival of *V. parahaemolyticus*. When the original concentration of *V. parahaemolyticus* in the VP^+^ water with crayfish was 5 × 10^2^ CFU/mL or 5 × 10^3^ CFU/mL, the *V. parahaemolyticus* could be continuously detected from 0.5 h to 4 h. When the *V. parahaemolyticus* concentration in the crayfish water rose to 5 × 10^4^ CFU/mL, the VP^+^ time was increased to at least 8 h.

**Table 5:** Survival of *V. parahaemolyticus* in water environments.

Original numbers of *V. parahaemolyticus* (CFU/mL)	Holding time for *V. parahaemolyticus* in water environments (h)									
	Pure water					Water containing crayfish				
	0.5	2	4	8	24	0.5	2	4	8	24
5 × 10^2^	−	−	−	−	−	+	+	+	−	−
5 × 10^3^	−	−	−	−	−	+	+	+	−	−
5 × 10^4^	+	+	−	−	−	+	+	+	+	−

–: *V. parahaemolyticus* negative. +: *V. parahaemolyticus* positive.

Sometimes, the for-sale crayfish were rinsed with water to clear away mud or restore their vitality. This case was also simulated in our study, where the crayfish were soaked in water containing different concentrations of *V. parahaemolyticus* for 0.5 h before detection. The results are shown in Table 6. When the concentration of *V. parahaemolyticus* in the rinse water was less than 5 × 10^2^ CFU/mL, all of the crayfish samples were VP^−^. When the *V. parahaemolyticus* concentration was greater than 5 × 10^2^ CFU/mL, the whole crayfish samples were VP^+^ at 0.5, 2, 4, and 8 h, while the CS samples were VP^+^ only at 0.5 h. When *V. parahaemolyticus* concentration was increased to 5 × 10^3^ CFU/mL or 5× 10^4^ CFU/mL, *V. parahaemolyticus* could be continuously detected in the CS samples from 0.5 h to 8 h or 24 h. Irrespective of the original concentration, the crayfish intestine was not immediately infected by the *V. parahaemolyticus*. Only when the *V. parahaemolyticus* concentration reached 5 × 10^4^ CFU/mL were the CI samples VP^+^ at 24 h. The *V. parahaemolyticus* contamination level was relatively lower in the crayfish continuously maintained in water than in the crayfish that were rinsed for a short time. This also indicated that fresh water was not conducive for *V. parahaemolyticus* spreading to crayfish.

**Table 6:** Results of the simulation tests of crayfish rinsed for 0.5 h with water carrying *V. parahaemolyticus.*

Original numbers of *V. parahaemolyticus* in the water for rinsing (CFU/mL)	Time after rinsing the crayfish with the water (h)														
	Whole crayfish					Crayfish surface					Crayfish intestine				
	0.5	2	4	8	24	0.5	2	4	8	24	0.5	2	4	8	24
5 × 10^0^	−	−	−	−	−	−	−	−	−	−	−	−	−	−	−
5 × 10^1^	−	−	−	−	−	−	−	−	−	−	−	−	−	−	−
5 × 10^2^	+	+	+	+	−	+	−	−	−	−	−	−	−	−	−
5 × 10^3^	+	+	+	+	+	+	+	+	+	−	−	−	−	−	−
5 × 10^4^	+	+	+	+	+	+	+	+	+	+	−	−	−	−	+

–: *V. parahaemolyticus* negative. +: *V. parahaemolyticus* positive.

### 3.5 The *Vibrio parahaemolyticus* contamination by the VP^+^ crayfish

The VP^+^ crayfish in the market may be another source of contamination of the VP^−^ crayfish for sale. In the simulation tests, we used pre-prepared VP^+^ crayfish to contaminate the VP^−^ crayfish. It was evident that the VP^+^ crayfish, in addition to the VP^+^ tank and VP^+^ water, could also contaminate the VP^−^ crayfish (Table 7). The original VP^−^ crayfish became VP^+^ when they contacted the VP^+^ crayfish for 0.5, 2, or 4 h. However, at 8 and 24 h, the crayfish became VP^−^ again. In addition, the positive detection results were only observed for the whole crayfish samples. It may be due to a low *V. parahaemolyticus* load in the pre-prepared VP^+^ crayfish. Similar results were observed in the simulation tests with the VP^+^ crayfish shells. The whole crayfish samples were VP^+^ when the VP^−^ crayfish contacted the VP^+^ crayfish shells for 0.5 or 2 h. This suggested that *V. parahaemolyticus* could spread from VP^+^ crayfish to VP^−^ crayfish by surface contact. Unfortunately, perhaps due to the small number of *V. parahaemolyticus* in the contamination source, the CS samples were not VP^+^ in either the simulations with the VP^+^ crayfish or in the simulations with the VP^+^ crayfish shells.

**Table 7:** Results of the simulation tests of contamination by *V. parahaemolyticus-*positive crayfish and crayfish shells.

*V. parahaemolyticus* source	Contact between crayfish and *V. parahaemolyticus* sources (h)														
	Whole crayfish					Crayfish surface					Crayfish intestine				
	0.5	2	4	8	24	0.5	2	4	8	24	0.5	2	4	8	24
VP^+(a)^ live crayfish	+	+	+	−	−	−	−	−	−	−	−	−	−	−	−
VP^+^ crayfish shell	+	+	−	−	−	−	−	−	−	−	−	−	−	−	−

^(a)^VP^+^ means *V. parahaemolyticus* positive. –: *V. parahaemolyticus* negative. +: *V. parahaemolyticus* positive.

### 3.6 The adsorption of *Vibrio parahaemolyticus* onto the crayfish shell

**Figure 1:**
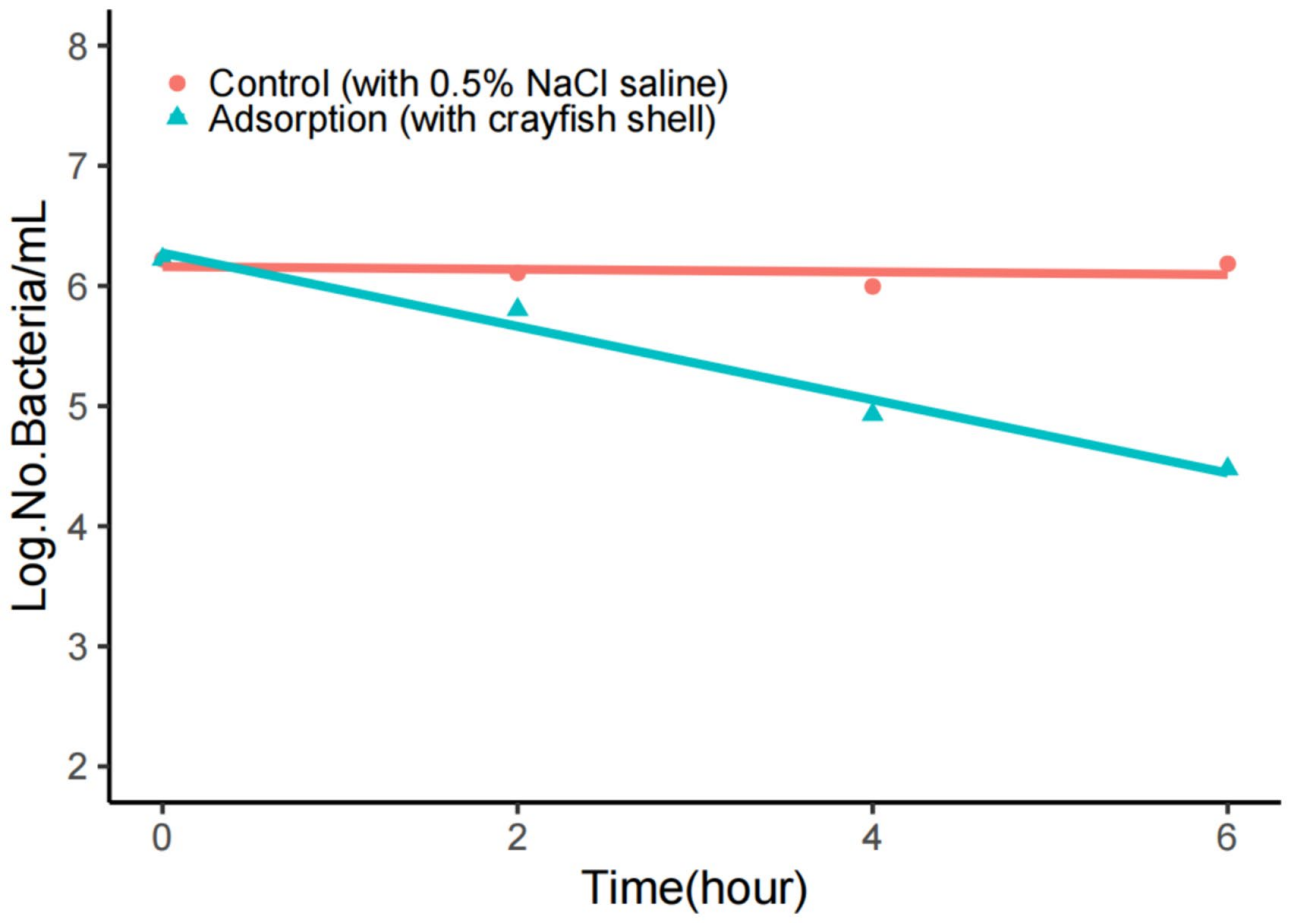
Adsorption of *V. parahaemolyticus* onto the crayfish shell. The adsorption buffer was 0.5% NaCl saline, and the pH was 7.0. The number of *V. parahaemolyticus* declined rapidly when adsorption occurred.

The shell comprises the main part of the crayfish surface. The simulation tests demonstrated that the VP^+^ crayfish shell could also be an important source of contamination. Here, the crayfish shell was placed in 0.5% NaCl saline to observe the adsorption of *V. parahaemolyticus*. The results are shown in Figure 1. In the filtrate samples that were generated by removing the crayfish shell from the mixture, the concentrations of *V. parahaemolyticus* declined exponentially, from 10^6^ CFU/mL to10^4^ CFU/mL in 6 h, while the concentrations of *V. parahaemolyticus* in the controls without crayfish shell were nearly unchanged during the 6-h period. The adsorption rate of *V. parahaemolyticus* onto the crayfish shell was >90% at 6 h, indicating a strong adsorption effect. The numbers of planktonic *V. parahaemolyticus* cells were also determined at longer adsorption times (8 and 24 h). However, the adsorption effect could not be evaluated due to significant bacterial growth or mass death (data not shown).

## 4 Discussion

### 4.1 Aquatic products markets are important *Vibrio parahaemolyticus* reservoirs

Fresh markets have been considered as reservoirs for many important food-associated pathogens. For the avian influenza virus, multiple genotypes were found in live bird markets, and the live bird market was reported to be one of the most important centers for viral recombination and evolution (Waziri et al., 2014; Youk et al., 2020). For foodborne pathogenic bacteria, the markets with products from different sources can preserve drug-resistant strains and genes (Wang et al., 2021; Guibert et al., 2023). Further, some foodborne pathogens exhibit partial preference in specific foods or circumstances in the markets. The important pathogen *V. cholerae* can colonize soft-shelled turtle surfaces, and it can easily be found in soft-shelled turtles for sale (Wang et al., 2017). Another study showed that different *Listeria monocytogenes* strains caused persistent contamination in different food stalls (Wang et al., 2022).

In many aquatic products markets, especially wholesale markets, various seafood and freshwater products are sold in the same space. It is well known that the seafood products (especially live seafood) often carry *V. parahaemolyticus* and inevitably introduce this pathogen to the markets. The *V. parahaemolyticus* numbers in gizzard shad and squid were up to 1,000 CFU/g and 10,000 CFU/g, respectively (Kim et al., 2017). The *V. parahaemolyticus* contamination level of oysters reached up to 10^7^ most probable number per gram (MPN/g) (Audemard et al., 2022). Meanwhile, several freshwater products could also introduce *V. parahaemolyticus* from farms to markets (Chen et al., 2021). Therefore, the *V. parahaemolyticus* strains could be isolated from both the seawater and freshwater products for sale in the markets (Pal and Das, 2010; Pei et al., 2016).

In addition to aquatic products, the VP^+^ environmental factors (such as the aquatic products containers and the water) are also important components of the *V. parahaemolyticus* reservoir in the markets. *V. parahaemolyticus* can form biofilms on the surfaces of various materials containing metal, glass, and plastic (Guo et al., 2020; Laverty et al., 2020; Leighton et al., 2023). It is not surprising that *V. parahaemolyticus* forms biofilm and persists in the environment of the aquatic products markets. In our study, we found that 19 out of 20 crayfish stall swabs were VP^+^, suggesting the persistence of *V. parahaemolyticus* in the aquatic products markets. In addition, incomplete cleaning of the mud containing *V. parahaemolyticus* cells (Pei et al., 2016) could also cause persistent contamination of *V. parahaemolyticus* in aquatic market stalls. In aquatic products markets, artificial seawater or fresh water are often used for storing or cleaning the products. Once the water is contaminated by *V. parahaemolyticus* sources, it could be involved in *V. parahaemolyticus* transmission. As seawater is optimal for the survival and growth of *V. parahaemolyticus*, the number of the bacterial cells in natural seawater can be up to 10^3^ MPN/mL (Audemard et al., 2022). Although fresh water is not suitable for the survival of *V. parahaemolyticus*, the bacteria could also be found in fresh water of some aquatic products stalls (Pei et al., 2016). In this study, one out of the 30 water samples from crayfish farms was VP^+^.

Thus, the markets selling aquatic foods are important reservoirs for *V. parahaemolyticus*. In general, the crayfish in the production and consumption chain are transferred from crayfish farms to wholesale markets, then from wholesale markets to retail markets, and finally from retail markets to consumers. As important reservoirs for *V. parahaemolyticus*, the wholesale and retail markets play important roles in spreading *V. parahaemolyticus* to crayfish.

### 4.2 Serious *Vibrio parahaemolyticus* contamination in crayfish occurs in the markets

With clinical data and epidemiological methods, crayfish had been recognized as a newly vehicle for *Vibrio* infections (Bean et al., 1998). But the *V. parahaemolyticus* contamination of crayfish before consumption was still unclear. In the present study, *V. parahaemolyticus* strains were isolated from both the crayfish for sale and the crayfish from farms, with the VP^+^ rates of 91 and 43%, respectively. In China, *V. parahaemolyticus* detection rate of aquatic products was 32% (Pang et al., 2020). Thus, serious *V. parahaemolyticus* contamination exists in the crayfish production-sale chain. Mok et al. (2021) found that the *V. parahaemolyticus* detection rate of a seawater shrimp, *Litopenaeus vannamei,* was 54.5% in the farming stage. Xu et al. (2014) found that the prevalence of *V. parahaemolyticus* in shrimps in Chinese retail market was 37.7%. Although the *V. parahaemolyticus* prevalence in crayfish may be lower than that of some seawater shrimp in farming stage, the *V. parahaemolyticus* detection rate in crayfish is significantly higher than in other shrimps in sale stage, indicating that selling in markets is the highest risk point for *V. parahaemolyticus* contamination in crayfish. No significant VP^+^ rate difference was found between the crayfish samples from wholesale markets and the samples from retail markets, implying that the *V. parahaemolyticus* contamination in crayfish reached saturation (a VP^+^ rate of nearly 90%) in a short time after the crayfish were placed in the markets.

The details concerning how *V. parahaemolyticus* contamination occurs in the crayfish for sale were also explored. Based on the detection results for the different parts of the crayfish, the VP^+^ rate of the CS samples (25/30) was slightly lower than that of the whole crayfish samples (30/30), while the VP^+^ rate of the CI samples (7/30) was significantly lower than those of the CS samples and the whole crayfish samples. The minor difference in VP^+^ rates between the whole crayfish samples and the CS samples may have been due to the different weights of samples used for the enrichment of *V. parahaemolyticus* (25 g samples for the whole crayfish and a swab for the CS samples). These results suggested that the surface of crayfish is an important target for *V. parahaemolyticus* contamination. This inference was supported by the contamination simulation tests, where the whole crayfish samples and the CS samples shared similar *V. parahaemolyticus* contamination trends. Many species in the *Vibrio* genus are part of the normal flora of marine organisms and endow them with biophysical functions such as bioluminescence (Milton, 2006). To date, there have been no reports of *V. parahaemolyticus* causing serious diseases in crayfish, and whether *V. parahaemolyticus* could colonize the crayfish intestine remains unknown. The VP^+^ rate of the CI samples implied that *V. parahaemolyticus* did not exist in most crayfish intestines. Further, the contamination simulation tests showed that the crayfish intestine was not easily contaminated by the various *V. parahaemolyticus* sources in the market environments.

Another important factor enhancing the *V. parahaemolyticus* contamination in crayfish for sale is the style of selling. As a vibrant crustacean, crayfish can survive for a long time when they are away from water environment. Therefore, most crayfish are stacked together in tanks without water in the processes of transport and sale. The continuous close contact between crayfish and VP^+^ crayfish tanks increases the risk of exposure to *V. parahaemolyticus*. The contamination simulations verified that the VP^+^ tank could easily contaminate the VP^−^ crayfish. Even tanks containing low numbers of *V. parahaemolyticus* could turn the VP^−^ crayfish to be VP^+^ in a short time. The simulation tests also confirmed that fresh water was harmful to *V. parahaemolyticus* survival and could more or less inhibit the *V. parahaemolyticus* contamination in crayfish. Although short-term (no more than 2 h) cleaning or soaking of crayfish with fresh water did not influence the *V. parahaemolyticus* contamination level, when the crayfish were kept in fresh water for longer time, the *V. parahaemolyticus* could be eliminated to a certain extent. Thus, the water-free sale style facilitates *V. parahaemolyticus* transmission and contamination in crayfish for sale.

### 4.3 The crayfish shell is an important target for *Vibrio parahaemolyticus* adsorption

In the VP^+^ market environments, the surface of crayfish is the first site of contact with *V. parahaemolyticus* sources. We tested the adsorption of *V. parahaemolyticus* onto the crayfish shell and found that the crayfish shell could efficiently adsorb *V. parahaemolyticus* cells within a short time, similar to copepods (Kaneko and Colwell, 1975). This suggested that the crayfish shell surface could capture *V. parahaemolyticus* from the contamination sources in the aquatic products markets. Chitin is a main component of the shell of marine organisms (Kaneko and Colwell, 1975; Younes and Rinaudo, 2015) as well as the freshwater crayfish. As an important carbon and nitrogen source, the metabolism of natural chitin has a significant impact on the carbon and nitrogen cycle (Kobayashi et al., 2023). Chitin is also involved in microbial biophysical factors such as biofilm formation (Markov et al., 2015) and bacterial competence (Blokesch, 2012; Cohen et al., 2021). *V. parahaemolyticus* carries a series of chitin-associated genes (Makino et al., 2003) and can use chitin as nutrient source (Hirano et al., 2019) for survival and growth. *V. parahaemolyticus* can express functional type IV pili to mediate its biofilm formation and adherence to chitin (Frischkorn et al., 2013; Williams et al., 2014; Billaud et al., 2022). The enzymatic hydrolysis products of chitin by *V. parahaemolyticus* can induce competence (Debnath and Miyoshi, 2022a). The induction is regulated by TfoX, CytR, and the quorum sensing system (Debnath and Miyoshi, 2022b). Based on previous studies of chitin function and our results in this study, we suspect that the chitin component of the crayfish shell is a crucial target for efficient adsorption of *V. parahaemolyticus*, and that this causes the high level of *V. parahaemolyticus* contamination in crayfish for sale. Further research is needed to verify the role of the crayfish shell in *V. parahaemolyticus* contamination and to explore the details of *V. parahaemolyticus* transmission.

## 5 Conclusion

**Figure 2:**
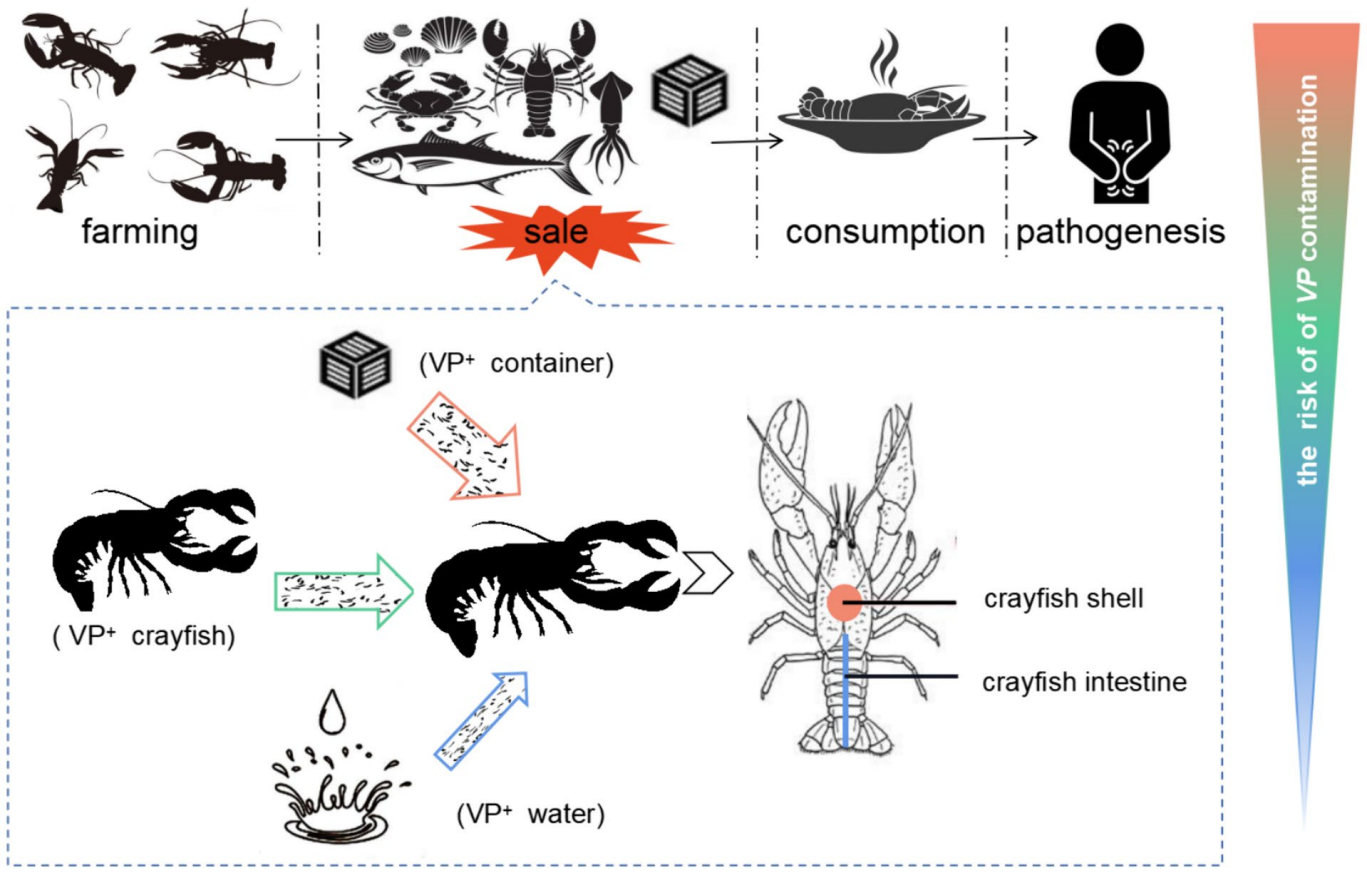
The contamination of *V. parahaemolyticus* in crayfish industry. The crayfish in the production and consumption chain are transferred from crayfish farms to aquatic markets, and from aquatic markets to consumers. The contamination of *V. parahaemolyticus* in the crayfish production-sale chain would threaten the consumers’ health. Because of the *V. parahaemolyticus-*rich background in aquatic markets and the water-free sale style of crayfish, the sale link has the highest risk for the contamination of *V. parahaemolyticus* in crayfish. In the sale link, various *V. parahaemolyticus* sources could contaminate the *V. parahaemolyticus*-free crayfish, while the VP^+^ crayfish containers contribute the most to the contamination. The crayfish shell is an important target for *V. parahaemolyticus* contamination. The crayfish uses the shell to acquire *V. parahaemolyticus* cells from the contamination sources and enhances the *V. parahaemolyticus* contamination of crayfish for sale. VP^+^ means *V. parahaemolyticus* positive.

Crayfish aquaculture is a large and rapidly growing industry associated with the national economy. The food-safety risk in crayfish industry is becoming serious. This study aimed to evaluate the level of *V. parahaemolyticus* contamination and identify high risk factors in the crayfish production-sale chain (Figure 2). We found that although there was serious *V. parahaemolyticus* contamination in all aspects of the crayfish production-sale chain, the sale stage contributed the most to the *V. parahaemolyticus* contamination. The crayfish containers in the market play the most important role in the *V. parahaemolyticus* contamination, while fresh water could inhibit the contamination. Further, the crayfish shell was confirmed to have strong adsorption capacity fo*r V. parahaemolyticus* and could easily capture the bacterium from *V. parahaemolyticus*-abundant market environments. Thus, the persistence of *V. parahaemolyticus* in aquatic products markets, the water-free sales style, and the high-efficiency adsorption are the main causes of the severe *V. parahaemolyticus* contamination in crayfish for sale. This study underscores the presence of *V. parahaemolyticus* contamination in crayfish for sale, and thus is of value for prevention and control the risk in the crayfish industry.

## Data availability statement

The original contributions presented in the study are included in the article/Supplementary material, further inquiries can be directed to the corresponding authors.

## Author contributions

KW: Conceptualization, Funding acquisition, Investigation, Methodology, Writing – original draft. DZ: Formal analysis, Investigation, Writing – original draft, Writing – review & editing. YL: Investigation, Methodology, Writing – review & editing. LX: Investigation, Methodology, Writing – review & editing. SS: Investigation, Methodology, Writing – review & editing. FT: Resources, Writing – review & editing. ML: Resources, Writing – review & editing. FG: Resources, Writing – review & editing. YZ: Validation, Writing – review & editing. XS: Conceptualization, Writing – review & editing. WW: Validation, Writing – review & editing. LW: Resources, Writing – review & editing. XN: Validation, Writing – review & editing. XZ: Project administration, Writing – review & editing. YF: Writing – review & editing. HL: Conceptualization, Supervision, Writing – review & editing.
