# Supplementary material for: Contamination of Vibrio parahaemolyticus in crayfish for sale
Source: Front Microbiol. 2024 Aug 14;15:1388658. doi: 10.3389/fmicb.2024.1388658 (PMC11349636; doi:10.3389/fmicb.2024.1388658)
Supplement: Supplementary file 1 [file Table_1.PDF]

Supplemental data for the manuscript "**Contamination of *Vibrio parahaemolyticus* in crayfish for sale**"

Kui Wu[1*#], Dazhao Zou[1, 2*], Yongyan Long[1], Lin Xue[1], Shufen Shuai[1], Feiyan Tian[3], Mei Li[4], Guoyin Fan[1], Yangyun Zheng[1], Xiangrong Sun[1], Wei Wang[1], Li Wang[1], Xiansheng Ni[1], Xiaoling Zhang[1], Yibing Fan[1], Hui Li[1#]

1 The Collaboration Unit for State Key Laboratory of Infectious Disease Prevention and Control, Jiangxi Provincial Health Commission Key Laboratory of Pathogenic Diagnosis and Genomics of Emerging Infectious Diseases, Nanchang Center for Disease Control and Prevention, Nanchang 330038, China

2 Jiangxi Provincial Key Laboratory of Preventive Medicine, School of Public Health, Jiangxi Medical College, Nanchang University, Nanchang 330006, China

3 Jiangxi Provincial Center for Agricultural Technical Extension, Nanchang 330046, China

4 Donghu District Center for Disease Control and Prevention, Nanchang 330008, China

[*] These authors contributed equally to this work.

[#] For correspondence. (K. Wu) E-mail: wooky07@163.com; Tel.: +86-79186363255; Fax: +86-79186363255 / (H. Li) E-mail: nccdcyjb@163.com; Tel.: +86-79186363255; Fax: +86-79186363255.

Table S1 The sampling information of this study

Number	Sample	Sampling date	Sample type	Source[1]	Sample location[2]
1	2023-M1	2023.05.08	Crayfish	LJFRM	Honggutan District, Nanchang City
2	2023-M2	2023.05.08	Crayfish	LJFRM	Honggutan District, Nanchang City
3	2023-M3	2023.05.08	Crayfish	LJFRM	Honggutan District, Nanchang City
4	2023-M4	2023.05.08	Crayfish	LJFRM	Honggutan District, Nanchang City
5	2023-M5	2023.05.08	Crayfish	LJFRM	Honggutan District, Nanchang City
6	2023-M6	2023.05.08	Crayfish	LJFRM	Honggutan District, Nanchang City
7	2023-M7	2023.05.10	Crayfish	CYRRM	Honggutan District, Nanchang City
8	2023-M8	2023.05.10	Crayfish	CYRRM	Honggutan District, Nanchang City
9	2023-M9	2023.05.10	Crayfish	CYRRM	Honggutan District, Nanchang City
10	2023-M10	2023.05.10	Crayfish	CYRRM	Honggutan District, Nanchang City
11	2023-M11	2023.05.29	Crayfish	GJWM	Donghu District, Nanchang City
12	2023-M12	2023.05.29	Crayfish	GJWM	Donghu District, Nanchang City
13	2023-M13	2023.05.29	Crayfish	GJWM	Donghu District, Nanchang City
14	2023-M14	2023.05.29	Crayfish	GJWM	Donghu District, Nanchang City
15	2023-M15	2023.05.29	Crayfish	GJWM	Donghu District, Nanchang City
16	2023-M16	2023.05.29	Crayfish	GJWM	Donghu District, Nanchang City
17	2023-M17	2023.05.29	Crayfish	GJWM	Donghu District, Nanchang City
18	2023-M18	2023.05.29	Crayfish	GJWM	Donghu District, Nanchang City
19	2023-M19	2023.05.29	Crayfish	GJWM	Donghu District, Nanchang City
20	2023-M20	2023.05.29	Crayfish	GJWM	Donghu District, Nanchang City
21	2023-M21	2023.06.19	Crayfish	LJFRM	onggutan District, Nanchang City
22	2023-M22	2023.06.19	Crayfish	LJFRM	onggutan District, Nanchang City
23	2023-M23	2023.06.19	Crayfish	LJFRM	onggutan District, Nanchang City
24	2023-M24	2023.06.19	Crayfish	LJFRM	onggutan District, Nanchang City
25	2023-M25	2023.06.19	Crayfish	LJFRM	onggutan District, Nanchang City
26	2023-M26	2023.06.19	Crayfish	LJFRM	onggutan District, Nanchang City
27	2023-M27	2023.07.04	Crayfish	CYRRM	onggutan District, Nanchang City
28	2023-M28	2023.07.04	Crayfish	CYRRM	onggutan District, Nanchang City
29	2023-M29	2023.07.04	Crayfish	CYRRM	onggutan District, Nanchang City
30	2023-M30	2023.07.04	Crayfish	LJFRM	onggutan District, Nanchang City
31	2023-M31	2023.07.04	Crayfish	LJFRM	onggutan District, Nanchang City
32	2023-M32	2023.07.04	Crayfish	LJFRM	onggutan District, Nanchang City
33	2023-M33	2023.07.04	Crayfish	LJFRM	onggutan District, Nanchang City
34	2023-M34	2023.07.04	Crayfish	LJFRM	onggutan District, Nanchang City
35	2023-M35	2023.07.04	Crayfish	LJFRM	onggutan District, Nanchang City
36	2023-M36	2023.07.04	Crayfish	LJFRM	onggutan District, Nanchang City
37	2023-M37	2023.07.18	Crayfish	GJWM	Donghu District, Nanchang City
38	2023-M38	2023.07.18	Crayfish	GJWM	Donghu District, Nanchang City
39	2023-M39	2023.07.18	Crayfish	GJWM	Donghu District, Nanchang City
40	2023-M40	2023.07.18	Crayfish	GJWM	Donghu District, Nanchang City
41	2023-M41	2023.07.18	Crayfish	GJWM	Donghu District, Nanchang City
42	2023-M42	2023.07.18	Crayfish	GJWM	Donghu District, Nanchang City
43	2023-M43	2023.07.18	Crayfish	GJWM	Donghu District, Nanchang City
44	2023-M44	2023.07.18	Crayfish	GJWM	Donghu District, Nanchang City
45	2023-M45	2023.07.18	Crayfish	GJWM	Donghu District, Nanchang City
46	2023-M46	2023.07.18	Crayfish	GJWM	Donghu District, Nanchang City
47	2023-M47	2023.07.25	Crayfish	LJFRM	Honggutan District, Nanchang City
48	2023-M48	2023.07.25	Crayfish	LJFRM	Honggutan District, Nanchang City
49	2023-M49	2023.07.25	Crayfish	LJFRM	Honggutan District, Nanchang City
50	2023-M50	2023.07.25	Crayfish	LJFRM	Honggutan District, Nanchang City
51	2023-M51	2023.07.25	Crayfish	LJFRM	Honggutan District, Nanchang City
52	2023-M52	2023.07.25	Crayfish	LJFRM	Honggutan District, Nanchang City
53	2023-M53	2023.07.25	Crayfish	LJFRM	Honggutan District, Nanchang City
54	2023-M54	2023.07.25	Crayfish	LJFRM	Honggutan District, Nanchang City
55	2023-M55	2023.07.31	Crayfish	GJWM	Donghu District, Nanchang City
56	2023-M56	2023.07.31	Crayfish	GJWM	Donghu District, Nanchang City
57	2023-M57	2023.07.31	Crayfish	GJWM	Donghu District, Nanchang City

58	2023-M58	2023.07.31	Crayfish	GJWM	Donghu District, Nanchang City
59	2023-M59	2023.07.31	Crayfish	GJWM	Donghu District, Nanchang City
60	2023-M60	2023.07.31	Crayfish	GJWM	Donghu District, Nanchang City
61	2023-M61	2023.07.31	Crayfish	GJWM	Donghu District, Nanchang City
62	2023-M62	2023.07.31	Crayfish	GJWM	Donghu District, Nanchang City
63	2023-M63	2023.07.31	Crayfish	GJWM	Donghu District, Nanchang City
64	2023-M64	2023.08.14	Crayfish	LJFRM	Honggutan District, Nanchang City
65	2023-M65	2023.08.14	Crayfish	LJFRM	Honggutan District, Nanchang City
66	2023-M66	2023.08.14	Crayfish	CYRRM	Honggutan District, Nanchang City
67	2023-M67	2023.08.14	Crayfish	CYRRM	Honggutan District, Nanchang City
68	2023-M68	2023.08.22	Crayfish	GJWM	Donghu District, Nanchang City
69	2023-M69	2023.08.22	Crayfish	GJWM	Donghu District, Nanchang City
70	2023-M70	2023.08.22	Crayfish	GJWM	Donghu District, Nanchang City
71	2023-M71	2023.08.22	Crayfish	GJWM	Donghu District, Nanchang City
72	2023-M72	2023.08.22	Crayfish	GJWM	Donghu District, Nanchang City
73	2023-M73	2023.08.22	Crayfish	GJWM	Donghu District, Nanchang City
74	2023-M74	2023.08.22	Crayfish	GJWM	Donghu District, Nanchang City
75	2023-M75	2023.08.22	Crayfish	GJWM	Donghu District, Nanchang City
76	2023-F1	2023.05.16	Crayfish	CF 1	Yujiang County, Yingtan City
77	2023-F2	2023.05.16	Crayfish	CF 2	Yujiang County, Yingtan City
78	2023-F3	2023.05.16	Crayfish	CF 3	Yujiang County, Yingtan City
79	2023-F4	2023.05.17	Crayfish	CF 4	Yugan County, Shangrao City
80	2023-F5	2023.05.17	Crayfish	CF 5	Yugan County, Shangrao City
81	2023-F6	2023.05.17	Crayfish	CF 6	Yugan County, Shangrao City
82	2023-F7	2023.05.17	Crayfish	CF 7	Jinxian County, Nanchang City
83	2023-F8	2023.05.17	Crayfish	CF 8	Jinxian County, Nanchang City
84	2023-F9	2023.05.17	Crayfish	CF 9	Jinxian County, Nanchang City
85	2023-F10	2023.05.16	Crayfish	CF 10	Duchang County, Jiujiang City
86	2023-F11	2023.05.16	Crayfish	CF 11	Duchang County, Jiujiang City
87	2023-F12	2023.05.16	Crayfish	CF 12	Duchang County, Jiujiang City
88	2023-F13	2023.05.17	Crayfish	CF 13	Pengze County, Jiujiang City
89	2023-F14	2023.05.17	Crayfish	CF 14	Pengze County, Jiujiang City
90	2023-F15	2023.05.17	Crayfish	CF 15	Pengze County, Jiujiang City
91	2023-F16	2023.05.17	Crayfish	CF 16	Yongxiu County, Jiujiang City
92	2023-F17	2023.05.17	Crayfish	CF 17	Yongxiu County, Jiujiang City
93	2023-F18	2023.05.17	Crayfish	CF 18	Yongxiu County, Jiujiang City
94	2023-F19	2023.05.30	Crayfish	CF 19	Jishui County, Jian City
95	2023-F20	2023.05.30	Crayfish	CF 20	Jishui County, Jian City
96	2023-F21	2023.05.30	Crayfish	CF 21	Jishui County, Jian City
97	2023-F22	2023.05.31	Crayfish	CF 22	Xingan County, Jian City
98	2023-F23	2023.05.31	Crayfish	CF 23	Xingan County, Jian City
99	2023-F24	2023.05.31	Crayfish	CF 24	Xingan County, Jian City
100	2023-F25	2023.05.30	Crayfish	CF 25	Wannian County, Shangrao City
101	2023-F26	2023.05.30	Crayfish	CF 26	Wannian County, Shangrao City
102	2023-F27	2023.05.30	Crayfish	CF 27	Wannian County, Shangrao City
103	2023-F28	2023.05.31	Crayfish	CF 28	Xinjian District, Nanchang City
104	2023-F29	2023.05.31	Crayfish	CF 29	Xinjian District, Nanchang City
105	2023-F30	2023.05.31	Crayfish	CF 30	Xinjian District, Nanchang City
106	2023-D1	2023.08.07	Tank swap	GJWM	Donghu District, Nanchang City
107	2023-D2	2023.08.07	Tank swap	GJWM	Donghu District, Nanchang City
108	2023-D3	2023.08.07	Tank swap	GJWM	Donghu District, Nanchang City
109	2023-D4	2023.08.07	Tank swap	GJWM	Donghu District, Nanchang City
110	2023-D5	2023.08.07	Tank swap	GJWM	Donghu District, Nanchang City
111	2023-D6	2023.08.07	Tank swap	GJWM	Donghu District, Nanchang City
112	2023-D7	2023.08.07	Tank swap	GJWM	Donghu District, Nanchang City
113	2023-D8	2023.08.07	Tank swap	GJWM	Donghu District, Nanchang City
114	2023-D9	2023.08.07	Tank swap	GJWM	Donghu District, Nanchang City
115	2023-D10	2023.08.07	Tank swap	GJWM	Donghu District, Nanchang City
116	2023-D11	2023.08.07	Tank swap	GJWM	Donghu District, Nanchang City

117	2023-D12	2023.08.07	Tank swap	GJWM	Donghu District, Nanchang City
118	2023-D13	2023.08.07	Tank swap	GJWM	Donghu District, Nanchang City
119	2023-D14	2023.08.07	Tank swap	GJWM	Donghu District, Nanchang City
120	2023-D15	2023.08.07	Tank swap	GJWM	Donghu District, Nanchang City
121	2023-D16	2023.08.07	Tank swap	GJWM	Donghu District, Nanchang City
122	2023-D17	2023.08.07	Tank swap	GJWM	Donghu District, Nanchang City
123	2023-D18	2023.08.07	Tank swap	GJWM	Donghu District, Nanchang City
124	2023-D19	2023.08.07	Tank swap	GJWM	Donghu District, Nanchang City
125	2023-D20	2023.08.07	Tank swap	GJWM	Donghu District, Nanchang City
126	2023-S1	2023.05.16	Water	CF 1	Yujiang County, Yingtan City
127	2023-S2	2023.05.16	Water	CF 2	Yujiang County, Yingtan City
128	2023-S3	2023.05.16	Water	CF 3	Yujiang County, Yingtan City
129	2023-S4	2023.05.17	Water	CF 4	Yugan County, Shangrao City
130	2023-S6	2023.05.17	Water	CF 6	Yugan County, Shangrao City
131	2023-S7	2023.05.17	Water	CF 7	Jinxian County, Nanchang City
132	2023-S8	2023.05.17	Water	CF 8	Jinxian County, Nanchang City
133	2023-S9	2023.05.17	Water	CF 9	Jinxian County, Nanchang City
134	2023-S10	2023.05.16	Water	CF 10	Duchang County, Jiujiang City
135	2023-S11	2023.05.16	Water	CF 11	Duchang County, Jiujiang City
136	2023-S12	2023.05.16	Water	CF 12	Duchang County, Jiujiang City
137	2023-S13	2023.05.17	Water	CF 13	Pengze County, Jiujiang City
138	2023-S14	2023.05.17	Water	CF 14	Pengze County, Jiujiang City
139	2023-S15	2023.05.17	Water	CF 15	Pengze County, Jiujiang City
140	2023-S16	2023.05.17	Water	CF 16	Yongxiu County, Jiujiang City
141	2023-S17	2023.05.17	Water	CF 17	Yongxiu County, Jiujiang City
142	2023-S18	2023.05.17	Water	CF 18	Yongxiu County, Jiujiang City
143	2023-S19	2023.05.30	Water	CF 19	Jishui County, Jian City
144	2023-S20	2023.05.30	Water	CF 20	Jishui County, Jian City
145	2023-S21	2023.05.30	Water	CF 21	Jishui County, Jian City
146	2023-S22	2023.05.31	Water	CF 22	Xingan County, Jian City
147	2023-S23	2023.05.31	Water	CF 23	Xingan County, Jian City
148	2023-S24	2023.05.31	Water	CF 24	Xingan County, Jian City
149	2023-S25	2023.05.30	Water	CF 25	Wannian County, Shangrao City
150	2023-S26	2023.05.30	Water	CF 26	Wannian County, Shangrao City
151	2023-S27	2023.05.30	Water	CF 27	Wannian County, Shangrao City
152	2023-S28	2023.05.31	Water	CF 28	Xinjian District, Nanchang City
153	2023-S29	2023.05.31	Water	CF 29	Xinjian District, Nanchang City
154	2023-S30	2023.05.31	Water	CF 30	Xinjian District, Nanchang City

1: GJWM: Ganjiang wholesale market. LJFRM: Laojiefang retail market. CYLRM: Cuiyuanlu retail market. CF: crayfish farm.

2 All the samples were collected in Jiangxi province, China.
